# Progerin cross-linking stiffens the nucleus and impairs mechanosensation in Hutchinson–Gilford progeria syndrome

**DOI:** 10.1242/jcs.264519

**Published:** 2026-06-19

**Authors:** Luv Kishore Srivastava, Ajinkya Ghagre, Ioannidis Duchastel-Vassaramva, Allen J. Ehrlicher

**Affiliations:** ^1^ Department of Bioengineering, McGill University, Montreal H3A 0E9, Canada; ^2^ School of Biomedical Engineering, University of British Columbia, Vancouver V6T 2B9, Canada; ^3^ Department of Biomedical Engineering, McGill University, Montreal H3A 2B4, Canada; ^4^ Department of Anatomy and Cell Biology, McGill University, Montreal H3A 0C7, Canada; ^5^ Centre for Structural Biology, McGill University, Montreal H3G 0B1, Canada; ^6^ Department of Mechanical Engineering, McGill University, Montreal H3A 0C3, Canada; ^7^ Rosalind and Morris Goodman Cancer Research Institute, McGill University, Montreal H3A 1A3, Canada

**Keywords:** Progerin, Nuclear mechanics, Lamin A/C, Chromatin, YAP, Hutchinson–Gilford progeria syndrome, Mechanotransduction, Nuclear wrinkling

## Abstract

Nuclear mechanosensation enables cells to detect and respond to mechanical cues through deformation governed by lamina and chromatin stiffness. In Hutchinson–Gilford progeria syndrome (HGPS), whether nuclear stiffening arises from progerin (a farnesylated variant form of lamin A) abundance alone and how it impacts mechanotransduction, remain unknown. Here, we show that progerin-driven cross-linking is the primary mechanical determinant of nuclear stiffness, and that this excessive stiffening impairs mechanosensation. We introduce a parameter called the cross-linked lamin expression factor (CLEF), which combines lamin abundance and immobile fraction as orthogonal components, and robustly predicts nuclear stiffness (*r*=0.84). Progerin aggregates tethered to the nuclear envelope induce persistent wrinkling resistant to hypotonic deformation, indicating stable mechanical anchoring. Strain mapping reveals that progerin spatial heterogeneity is a stronger local stiffness determinant than lamin A/C, and chromatin decompaction in HGPS makes progerin-rich lamina regions the dominant mechanical contributors. Excessive lamina cross-linking impairs YAP (also known as YAP1) nuclear translocation across multiple independent perturbations of lamina mechanics, disrupting mechanosensation. These findings provide a mechanistic framework connecting nuclear architecture to defective mechanotransduction in laminopathies.

## Teaser

**Summary:** Progerin tethers irreversibly into the nuclear lamina in progeria, over-stiffening the nucleus and silencing the mechanical signals that cells depend on to function.

## INTRODUCTION

The nucleus is a crucial organelle in eukaryotic cells that houses the DNA and integrates both biochemical and mechanical signals to regulate gene expression and cell function (Lamond and Earnshaw, 1998). Beyond its genetic role, the nucleus is increasingly recognised as a central mechanosensory structure capable of responding to mechanical cues (Kirby and Lammerding, 2018). Nuclear mechanotransduction refers to the process by which the nucleus detects mechanical inputs and converts them into biochemical signals that regulate cellular behaviour.

With an effective Young's modulus ranging from ∼1 to 10 kPa (Vaziri and Mofrad, 2007; Caille et al., 2002), the nucleus is considerably stiffer than the surrounding cytoplasm (∼10 Pa) (Guo et al., 2013). The nuclear envelope, composed of a double lipid bilayer supported by the nuclear lamina, serves as both a physical barrier and a structural scaffold. Disruptions in nuclear morphology can impair force transmission and interfere with proper mechanosensation, contributing to various disease states, such as cancers and ageing-related laminopathies (Lammerding et al., 2005; Janin et al., 2017; Worman et al., 2010). The nuclear lamina is composed primarily of lamin A/C and lamin B isoforms, with lamin A/C playing a dominant role in determining both global and local nuclear mechanics (Dechat et al., 2010; Lammerding et al., 2006; Lammerding, 2011; Srivastava et al., 2021). Mutations or abnormal processing of lamin A/C, as seen in laminopathies such as Hutchinson–Gilford Progeria Syndrome (HGPS or progeria), can significantly alter nuclear stiffness and structure (Capo-Chichi et al., 2011; Verstraeten et al., 2008; Dahl et al., 2006). In progeria, a point mutation in LMNA results in the production of a permanently farnesylated variant of lamin A, known as progerin, which accumulates aberrantly at the nuclear envelope (Young et al., 2006; Lombardi and Lammerding, 2010; Pollex and Hegele, 2004).

Previous studies using micropipette aspiration and substrate stretch have shown that progeria nuclei are more rigid than their wild-type counterparts, both in isolated and intact cells (Verstraeten et al., 2008; Dahl et al., 2006; Apte et al., 2017). However, the precise mechanical changes in progeria nuclei and their contribution to cellular dysfunction remain poorly understood. Because nuclear mechanics influence both chromatin architecture and the trafficking of mechanosensitive transcription factors, such as YAP (also known as YAP1) (Damodaran et al., 2018; Seelbinder et al., 2021; Kalukula et al., 2022; Elosegui-Artola et al., 2017), alterations in nuclear stiffness might profoundly affect cell fate and function. Furthermore, the spatial distribution of lamin A/C and progerin can introduce heterogeneity in local nuclear stiffness, potentially altering mechanical signal transmission at sub-micron scales (Srivastava et al., 2021).

These mechanical alterations are particularly important in the context of chromatin dynamics and gene regulation. Epigenetic changes, including chromatin condensation and histone modifications, can be both a cause and consequence of altered nuclear mechanics (Klutstein et al., 2016; Kulis and Esteller, 2010; Moosavi and Motevalizadeh Ardekani, 2016). In progeria, chromatin structure is disrupted, and heterochromatin is often reduced (Shumaker et al., 2006; Chojnowski et al., 2020), further contributing to impaired mechanosensation. These changes may lead to irregular force transmission, unbalanced mechanosensitive pathways like YAP/TAZ (TAZ is also known as WWTR1) signalling, and transcriptional dysregulation (Damodaran et al., 2018; Kalukula et al., 2022; Moosavi and Motevalizadeh Ardekani, 2016).

Although it is established that progerin expression increases nuclear stiffness (Booth et al., 2015), whether this arises from increased protein abundance alone or from altered lamina cross-linking dynamics has not been determined. Furthermore, the downstream consequences of this stiffening for nuclear mechanosensory function remain unknown. We hypothesise that progerin-driven cross-linking is the primary mechanical determinant of nuclear stiffness in HGPS, and that this excessive stiffening impairs mechanosensation through the Hippo-YAP pathway.

In this study, we address these gaps by systematically investigating how progerin cross-linking alters nuclear mechanics and mechanosensation in progeria fibroblasts. We introduce the cross-linked lamin expression factor (CLEF) to quantitatively capture functional lamina cross-linking and its contribution to nuclear stiffness. We further demonstrate that progerin forms intranuclear aggregates that mechanically tether to the nuclear envelope, driving persistent nuclear wrinkling. By combining high-resolution strain mapping with chromatin condensation analysis, we uncover that in progeria, reduced heterochromatin condensation weakens the mechanical contribution of chromatin, leaving cross-linked progerin-rich lamina regions as the dominant determinant of nuclear stiffness. Finally, we reveal that these mechanical and structural alterations impair YAP-mediated mechanotransduction, providing mechanistic insight into how nuclear architecture dysfunctions contribute to signalling defects in progeria. This study elucidates the qualitative impact of lamin cross-linking, introduces the CLEF framework for mechano-stiffness prediction, and identifies intranuclear progerin tethering as a novel mechanism impairing nuclear deformability and mechanotransduction.

## RESULTS

### Progeria nuclei are stiffer than wild-type human dermal fibroblast cell nuclei

**Fig. 1.**
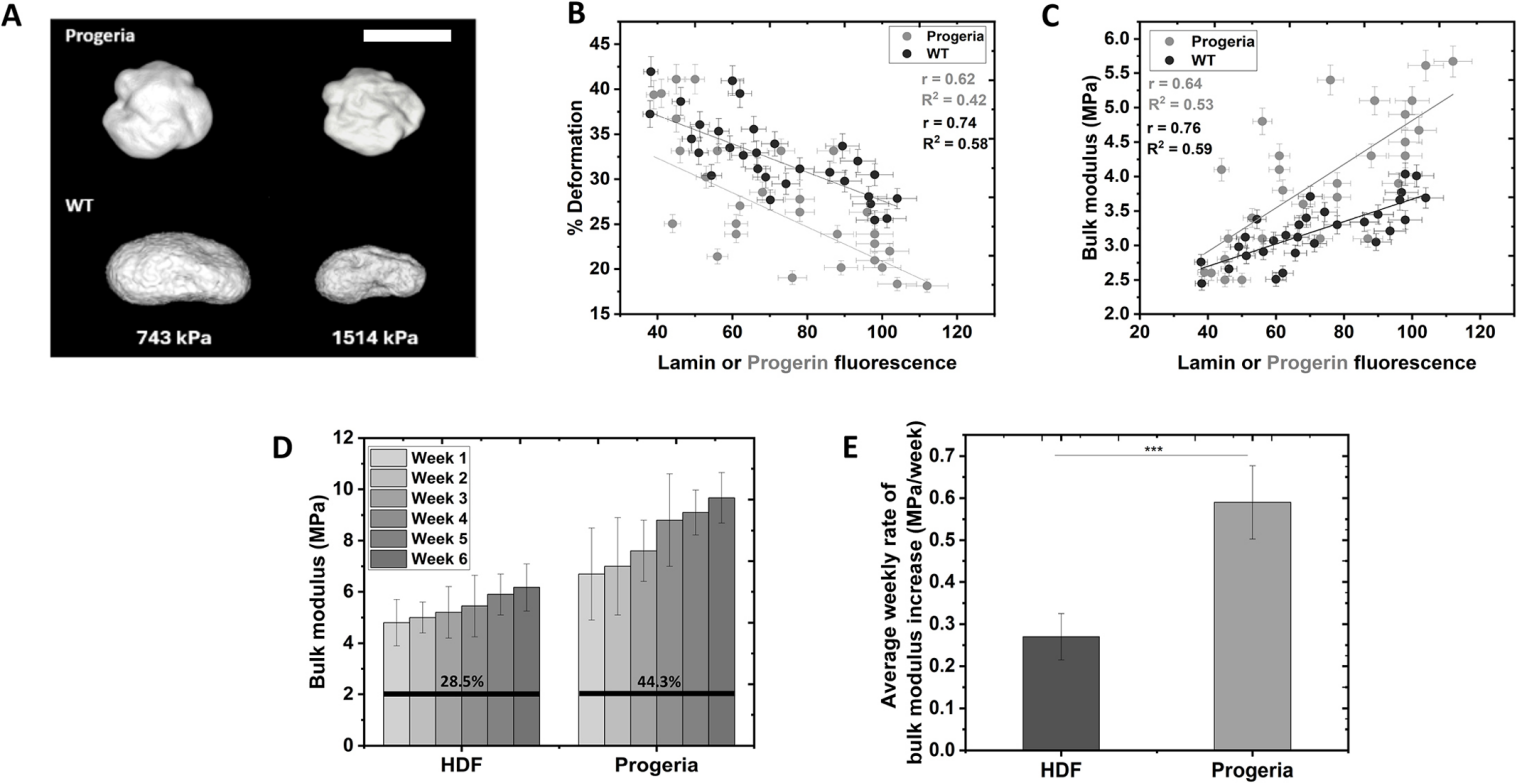
**Hyperosmotic compression of nuclei in cells.** (A) 3D reconstructions of representative WT and progeria nuclei before (isotonic medium; 743 kPa) and after hyperosmotic compression (10% PEG 400; ΔP=771 kPa). Images of a single progeria and WT nucleus before and after hyperosmotic compression, taken from a single experiment. Scale bar: 5 μm. (B) Nuclear deformation in silenced WT cells transfected with lamin A/C or progerin showing that deformation decreases as protein expression increases, and progerin-transfected cells are less deformed than WT cells at comparable expression levels (*n*=30 per cell type from two independent experiments; Pearson r: Progeria=0.62, R²=0.42; WT=0.74, R²=0.58; error bars represent propagated measurement error). (C) Bulk moduli of silenced WT cells transfected with lamin A/C or progerin showing that stiffness increases as protein expression increases, and progerin-transfected cells are stiffer than WT cells at comparable expression levels (*n*=30 per cell type from two independent experiments; Pearson r: Progeria=0.64, R²=0.53; WT=0.76, R²=0.59; error bars represent propagated measurement error). Across the overlapping fluorescence range, progerin-expressing nuclei are consistently less deformed (B) and stiffer (C) than lamin A/C-expressing nuclei, demonstrating that the stiffness difference is not solely dependent expression level. (D) Nuclear stiffening over 6 weeks in progeria nuclei compared to WT nuclei illustrating that progeria nuclei begin at a higher relative stiffness and stiffen more rapidly (n=22 per cell type from two independent experiments, mean±s.d.). (E) The average weekly rate of bulk modulus increase was significantly greater in progeria than in WT fibroblasts (progeria: 0.639±0.057 MPa/week versus WT: 0.280±0.021 MPa/week; ANCOVA, t=5.910, d.f.=8, *P*=0.0004; mean±s.d.). ***P<0.001.

To measure the stiffness of the nuclei as a function of progerin or lamin A/C expression, we first knocked down endogenous lamin A/C in healthy wild-type human dermal fibroblasts (WT) using an RFP-tagged inducible shRNA construct targeting lamin A/C (Dharmacon; cat. #V3THS_352819), reducing its contribution to nuclear mechanics (Fig. S1). At 24 h after lamin A/C silencing, the silenced WT cells were then transfected with mCherry-tagged wild-type lamin A/C or EGFP-tagged lamin A D50 mutant plasmid (progeria-causing mutation) and imaged 24 h post transfection. To quantify the bulk modulus of the nuclei, cells were exposed to an osmotic pressure of 1514 kPa using 10% 400 Da polyethylene glycol (PEG) in culture medium (net stress on the cell 1514–743=771 kPa). PEG creates a hyperosmotic environment, leading to water expulsion and nuclear compression (Srivastava et al., 2021; Khavari and Ehrlicher, 2019) (Fig. 1A).

The nuclear deformation under osmotic compression decreased with an increase in lamin A/C or progerin expression, with deformation larger with WT lamin A/C compared to that seen in progerin-containing nuclei (Fig. 1B). This was also reflected in the nuclear bulk moduli, where cells expressing more lamin A/C or progerin are relatively stiffer, as reported previously (Srivastava et al., 2021), but progerin-expressing nuclei were found to be substantially stiffer than wild-type lamin A/C with a larger bulk modulus (Fig. 1C).

Given that HGPS fibroblasts accumulate progerin progressively with passage (Goldman et al., 2004), leading to increasing nuclear stiffness, we next compared the bulk moduli of unmodified WT (Coriell Institute cat. #GM03234; male) and HGPS (Coriell Institute cat. #AG11498; male) fibroblast cells as a function of time in culture. We note that replicative passaging is an imperfect model of physiological ageing as senescence-associated changes might not fully recapitulate the *in vivo* ageing trajectory. Nevertheless, passage-dependent stiffening provides a tractable readout for monitoring mechanical deterioration in both healthy and progeria fibroblasts. We also acknowledge that the WT donor (GM03234, 23 years) and HGPS donor (AG11498, 14 years) differ in age and donor-to-donor variation in baseline lamin expression cannot be fully excluded. The initial moduli of progeria nuclei at passage number 13 (6.7±1.8 MPa) were found to be larger than those of normal WT nuclei (4.8±0.9 MPa) with the same passage number. In our study, we discovered that the bulk moduli of both types of cells increased over the course of 6 weeks in culture (Fig. 1D). However, the rate of passage-dependent nuclear stiffening was substantially higher in HGPS than in WT fibroblasts (Fig. 1E).

### Reduced lamin A mobility explains increased nuclear stiffness in progeria cells

As shown in Fig. 1B,C, nuclei from progeria cells exhibit markedly higher stiffness than those from WT cells, despite having similar levels of total lamin fluorescence intensity (lamin A/C in WT cells and progerin in progeria cells). Interestingly, even among progeria cells with comparable progerin fluorescence, we observed substantial variability in nuclear stiffness, suggesting that expression levels alone do not fully account for mechanical differences. This led us to hypothesise that lamin A/C cross-linking, rather than simple abundance, might underlie the increased and heterogeneous nuclear stiffness in progeria. We reasoned that cross-linking would stiffen the nucleus through diverse mechanisms, including entropic restrictions and tightening the mesh, which stiffens with increased cross-linking.

**Fig. 2.**
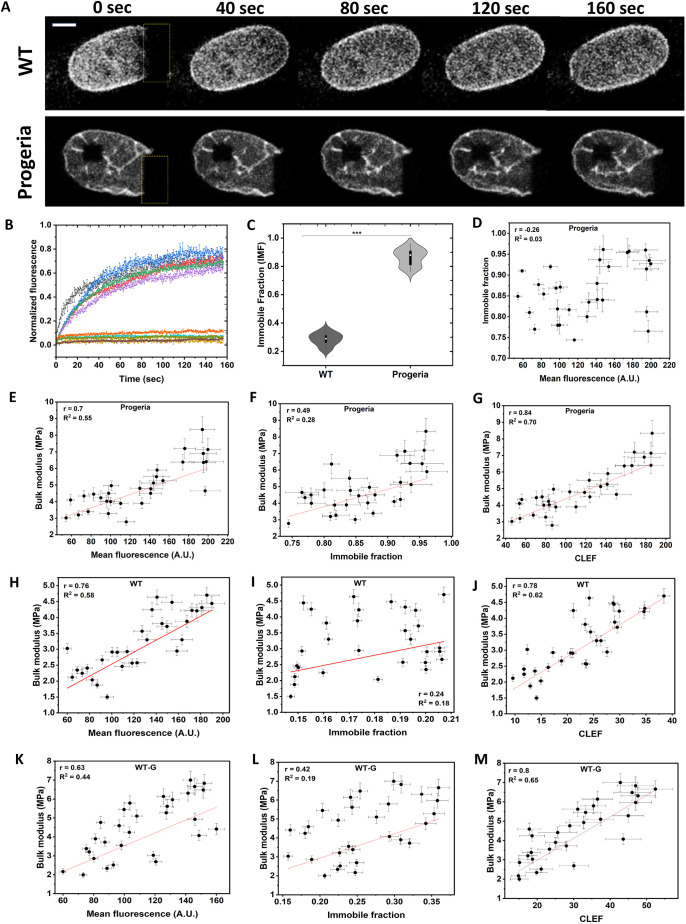
**Progerin expression reduces lamin mobility at the nuclear envelope.** (A) Representative time-lapse confocal images showing FRAP of lamin A in WT (top row) and progerin in progeria (bottom row) nuclei. A defined region at the nuclear periphery (yellow box) was photobleached and fluorescence recovery was monitored over time. Scale bar: 3 μm. (B) Representative FRAP recovery curves from WT and progeria nuclei showing significantly attenuated fluorescence recovery in progeria (*n*=5). (C) Quantification of immobile fraction (IMF) reveals significantly impaired lamin dynamics in progeria compared to WT [violin plots with a box plot in the middle highlighting median (centre dot), interquartile range (box), and 1.5× IQR from the Q1 and Q3 boundaries (whiskers); *n*=30 nuclei from three independent experiments]. ***P<0.001 (unpaired two-tailed *t*-test). (D) The immobile fraction of progerin is independent of its expression level (Pearson r=−0.26, R²=0.03; *n*=30 nuclei from three independent experiments), establishing that the two CLEF components carry orthogonal information. (E) Total progerin fluorescence alone shows a moderate positive correlation with bulk modulus (r=0.70, R²=0.55; Progeria; *n*=30 nuclei from three independent experiments). (F) The progerin immobile fraction alone shows a weaker correlation with bulk modulus (r=0.49, R²=0.28, *P*<0.001; progeria; *n*=30 nuclei from three independent experiments). (G) The CLEF (fluorescence×immobile fraction) shows a markedly stronger correlation with bulk modulus than either component alone (r=0.84, R²=0.70, *P*<0.001; progeria; *n*=30 nuclei from three independent experiments), demonstrating that nuclear stiffness is governed jointly by progerin abundance and cross-linking state. (H–J) Equivalent analysis in unmodified WT cells: fluorescence alone (r=0.76, R²=0.58; H), immobile fraction alone (r=0.24, R²=0.18; I) and CLEF (r=0.78, R²=0.62; J). CLEF offers minimal improvement over fluorescence alone in WT, consistent with uniformly low cross-linking state variance (*n*=30 nuclei from three independent experiments). (K–M) Equivalent analysis in WT cells treated with GGTI (WT-G), where geranylgeranyltransferase I inhibition pharmacologically increases lamin A/C immobility without progerin: fluorescence alone (r=0.63, R²=0.44; K), immobile fraction alone (r=0.42, R²=0.19; L) and CLEF (r=0.80, R²=0.65; M; *n*=30 nuclei from three independent experiments). CLEF outperforms both components in this progerin-free context, validating the framework independently. Error bars in D–M represent propagated measurement error. A.U., arbitrary units.

To test whether differences in lamin A/C or progerin mobility contribute to nuclear stiffness, we performed fluorescence recovery after photobleaching (FRAP) on WT cells expressing mCherry–lamin A/C or GFP–progerin in progeria cells. WT nuclei displayed substantial fluorescence recovery after bleaching, consistent with a highly mobile lamin A/C population. In contrast, progeria nuclei exhibited greatly reduced fluorescence recovery over time (Fig. 2A,B), indicating a significantly higher immobile fraction of progerin (Fig. 2C). This suggests that in progeria, progerin is more stably integrated into the nuclear lamina, likely through farnesyl-mediated enhanced cross-linking or membrane anchoring.

Chang et al. (2019) have demonstrated reduced mobility of linker of nucleoskeleton and cytoskeleton (LINC) complex components (nesprin-2G, SUN2 and emerin) in HGPS with partial restoration by farnesyltransferase inhibition, but that study did not include direct FRAP measurements of lamin A/C or progerin itself. Scaffidi and Misteli (Scaffidi and Misteli, 2005) have shown that WT GFP–lamin A exhibits reduced FRAP recovery kinetics in HGPS cells compared to controls, indicating that progerin immobilises even WT lamin A in a dominant-negative manner – an effect reversed upon elimination of progerin by splicing correction. Our study provides a complementary contribution by directly quantifying progerin mobility and linking mobility changes quantitatively to nuclear stiffness via the defining the CLEF. Lamin A/C and progerin were tagged with different fluorophores (mCherry and GFP, respectively); absolute numerical comparisons of mobile fractions across constructs were therefore avoided. Instead, intra-construct analyses and normalised recovery kinetics within each fluorophore channel were used, ensuring that observed differences in immobile fractions reflect biological rather than fluorophore-related factors. The mechanistic basis for farnesylation-dependent progerin immobilisation is well supported. Capell et al. (2005) have shown that non-farnesylated progerin fails to associate stably with the nuclear envelope, and Dechat et al. (2007) have demonstrated that the permanent farnesyl group anchors progerin constitutively to the inner nuclear membrane, preventing normal turnover. Furthermore, we found that lonafarnib increased progerin mobility specifically in progeria cells but not in WT cells treated with the same drug, consistent with farnesyl-mediated anchoring as the mechanism rather than a general drug effect (Fig. S2). To evaluate how reduced lamin mobility relates to nuclear stiffness, we first examined whether progerin abundance and the immobile fraction carry independent information. The immobile fraction of progerin showed no significant correlation with its fluorescence intensity [Pearson's r=−0.26, R²=0.03, *P*=0.16 (n.s.); Fig. 2D], indicating that how cross-linked progerin does not simply scale with how much is present. This orthogonality means that combining the two variables captures information that neither alone provides. Progerin fluorescence alone showed a moderate positive correlation with bulk modulus (r=0.70, R²=0.55, *P*<0.001; Fig. 2E) but left substantial variance unexplained. The immobile fraction alone showed an even weaker relationship (r=0.49, R²=0.28, *P*<0.001; Fig. 2F). We therefore defined the CLEF as the product of total progerin fluorescence and immobile fraction per nucleus. Because these quantities are orthogonal, their product integrates protein abundance with cross-linking state into a single composite metric. CLEF showed a markedly stronger correlation with bulk modulus than either component alone (Pearson's r=0.84, R²=0.70, *P*<0.001; Fig. 2G), demonstrating that nuclear stiffness in HGPS is governed jointly by how much progerin is present and how immobilised it is within the lamina network.

To assess whether the CLEF framework generalises beyond progerin-expressing cells, we examined its predictive power in two independent contexts. First, in unmodified WT cells, where lamin A/C cross-linking varies naturally across the population, we found that fluorescence alone correlated only moderately with bulk modulus (r=0.76, R²=0.58; Fig. 2H), whereas the immobile fraction showed a much weaker relationship (r=0.24, R²=0.18; Fig. 2I), consistent with WT lamin A/C being more dynamically integrated into the lamina. Nevertheless, the CLEF did not have a substantially improved predictive power over fluorescence alone (r=0.78, R²=0.62 versus r=0.76, R²=0.58; Fig. 2J), suggesting that in WT cells lamin A/C abundance is itself the primary mechanical determinant of nuclear stiffness, with cross-linking state contributing comparatively little – consistent with the dynamic, mobile nature of WT lamin A/C under basal conditions. Second, to test the utility of CLEF under a pharmacological perturbation that increases lamin A/C anchoring independently of progerin, we treated WT cells with geranylgeranyltransferase I inhibitor (GGTI, denoted WT-G), which blocks an alternative prenylation pathway. This intervention prevents the final proteolytic cleavage typically mediated by ZMPSTE24, causing a constitutive accumulation of prenylated prelamin A (Chang et al., 2012) in which a hydrophobic lipid group ‘farnesyl’ remains attached to the prelamin A and promotes aberrant membrane retention of lamin A/C, partially mimicking the permanently farnesylated state of progerin (Moiseeva et al., 2016). In WT-G cells, fluorescence alone showed a moderate correlation with the bulk modulus (r=0.63, R²=0.44; Fig. 2K), and the immobile fraction alone a weaker one (r=0.42, R²=0.19; Fig. 2L). The CLEF again outperformed both components (r=0.80, R²=0.65; Fig. 2M), confirming that the composite metric captures the mechanical contribution of cross-linking regardless of whether anchoring arises from progerin farnesylation or pharmacological inhibition of lamin turnover.

Together, these findings reveal that the mechanical phenotype in progeria is not solely driven by lamin A abundance, but is crucially shaped by its cross-linked, immobile fraction, likely enhanced by progerin farnesylation. Across all three contexts, CLEF maintained a strong correlation with nuclear stiffness, outperforming either component alone. Notably, the degree to which cross-linking augmented predictive power was context dependent – in progeria and WT-G cells, the immobile fraction substantially increased predictive power over fluorescence alone, whereas in healthy WT cells, lamin A/C abundance was already the dominant predictor and cross-linking contributed comparatively little. We hypothesise that progerin-driven cross-linking is the primary mechanical determinant of nuclear stiffness in HGPS, and that this excessive stiffening impairs mechanosensation through the YAP pathway and potentially others. The consistent performance of the CLEF across WT, WT-G and progeria contexts demonstrates that it captures a generalisable mechanical principle of lamin organisation spanning physiological to pathological cross-linking states.

### Progeria nuclei are wrinkled due to internal progerin structures

**Fig. 3.**
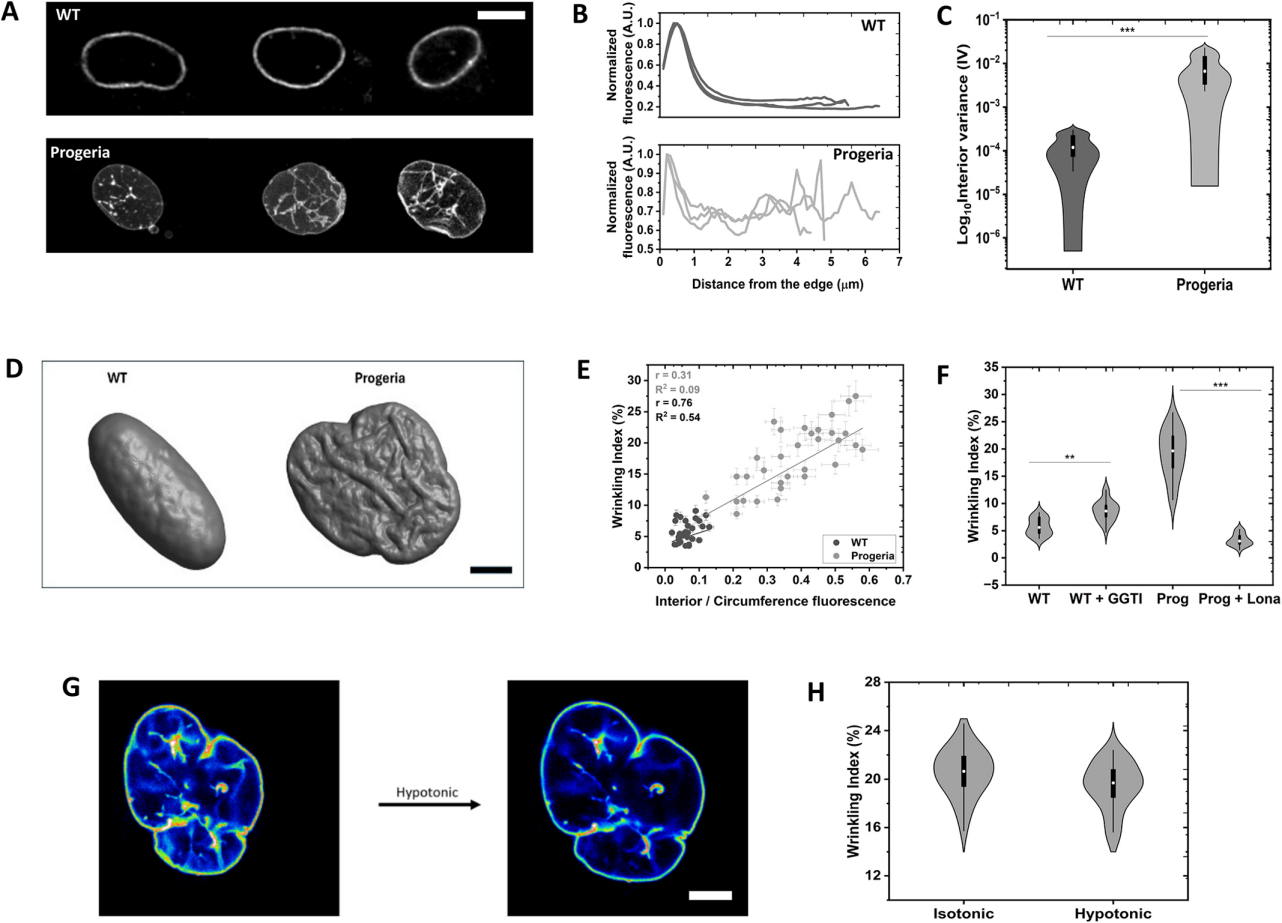
**Nuclear wrinkling in progeria cells.** (A) Representative images showing lamin A/C fluorescence in WT cells (top) and progerin fluorescence in progeria cells. Scale bar: 5 μm. (B) Ring-based radial profiles of the representative images in A reveal a steep peripheral drop-off in WT versus irregular, elevated interior signal in progeria nuclei. (C) Interior variance (IV) is significantly higher in progeria, reflecting increased high-frequency undulations in the interior profile (*n*=30 for each cell type from three independent experiments). ***P<0.001 (unpaired two-tailed t-test). (D) 3D reconstructs of WT and progeria nucleus showing wrinkling. Scale bar: 3 μm. (E) The WI was found to increase with an increase in the progerin fluorescence inside the nucleus, but this relationship was not observed for WT nuclei (*n*>30 for each cell from three independent experiments, error bars represent propagated measurement error). Fisher's z-test confirmed a significantly stronger correlation between nuclear wrinkling and interior protein localisation in progeria (r=0.76) than in WT (r=0.31; z=2.26, *P*=0.024). (F) GGTI treatment increases and lonafarnib treatment decreases WI in WT and progeria cells respectively (*n*=30 for each cell from three independent experiments). **P<0.01; ***P<0.001 (one-way ANOVA with Tukey's post hoc test). (G) Representative progeria nuclei before and after hypotonic swelling. Scale bar: 3 μm. (H) No statistical significance between the WI in isotonic or hypotonic medium (*n*=30 for each condition from three independent experiments). Data in C, F and H shown as violin plots with a box plot in the middle highlighting median (centre dot), interquartile range (box), and 1.5× IQR from the Q1 and Q3 boundaries (whiskers). A.U., arbitrary units.

Given the abnormal incorporation of progerin into nuclear lamina, we examined its spatial distribution in progeria cells relative to WT. In WT nuclei, lamin A/C was localised predominantly to the nuclear periphery, whereas in progeria nuclei progerin also formed filamentous networks and punctate accumulations throughout the nucleoplasm (Fig. 3A). To quantify these differences, we performed radial profiling on sum-of-slices *z*-projections, measuring mean fluorescence at the nuclear membrane and then stepwise at 0.1-μm intervals toward the nuclear interior using concentric rings. This analysis revealed a steep decline in lamin A/C intensity from the periphery inward in WT cells, whereas progerin in progeria nuclei remained elevated and irregularly distributed throughout the nucleoplasm (Fig. 3B). Correspondingly, quantification of fluorescence variance across the interior (interior variance; IV) demonstrated significantly greater heterogeneity in progeria nuclei compared to that in WT, consistent with the presence of internal progerin aggregates (Fig. 3C).

Aligned with this altered distribution, progeria nuclei displayed pronounced nuclear envelope wrinkling compared to WT cells (Fig. 3D). We quantified nuclear wrinkling of lamin A/C or progerin fluorescence across the nuclear periphery using the wrinkling index (WI) parameter, as described in the Materials and Methods section (Cosgrove et al., 2021). This method captures variations in envelope topography, where higher WI values indicate more wrinkled nuclear surfaces. Progeria nuclei exhibited substantially elevated WI values, suggesting that internal progerin networks exert inward tethers on the lamina, deforming the nuclear envelope (Fig. S3A).

Quantitative analysis revealed that the proportion of intranuclear progerin structures relative to the nuclear periphery positively correlated with the degree of nuclear wrinkling, as measured by the WI (Fig. 3E), reinforcing the role of internal progerin accumulations in deforming the nuclear envelope. As described above, GGTI treatment was employed here to increase lamin A/C immobility by blocking the alternative prenylation of prelamin A. By forcing the retention of farnesyl, the lamina becomes irreversibly tethered to the nuclear envelope, providing a pharmacological model of lamina hyper-stabilisation and stiffness independent of progerin expression (Park et al., 2025). GGTI increased the proportion of intranuclear lamin A/C invaginations in WT cells whereas lonafarnib treatment reduced intranuclear progerin invaginations in progeria cells (Fig. S3B). These pharmacological perturbations confirm that the spatial redistribution of lamin proteins between the nuclear periphery and the nucleoplasm is governed by prenylation status, and that this redistribution directly modulates the degree of nuclear envelope wrinkling. Consistent with this model, pharmacological perturbation of prenylation altered wrinkling – GGTI treatment increased WI in WT cells from 5.7±1.6% to 9.2±1.8%, whereas lonafarnib reduced WI in progeria cells 18.7±4.8% to 3.3±1.1% (mean±s.d.; Fig. 3F). We reasoned that if these internal progerin structures are mechanically tethered to nuclear wrinkles, swelling the nucleus would not alleviate wrinkling. To test this, we exposed cells to hypotonic medium to induce nuclear swelling and observed no significant changes in the WI of progeria cells (Fig. 3G,H), supporting the notion of stable mechanical anchoring between internal progerin networks and nuclear lamina. These findings highlight that the abnormal internal aggregation of progerin in progeria cells, contrasting with the peripheral localisation of lamin A/C in WT cells, is a key driver of nuclear wrinkling. This altered spatial distribution of progerin likely affects the mechanical integrity of the nuclear lamina, which is known to influence chromatin organisation, gene expression and overall nuclear stiffness (Columbaro et al., 2005; Goldman et al., 2004). Previous studies have shown that increased nuclear wrinkling impairs chromatin dynamics and accessibility, potentially disrupting transcriptional regulation and genomic stability in progeria cells (Kim et al., 2024). Our data suggests that targeting the aberrant internal aggregation of progerin could serve as a therapeutic strategy to alleviate nuclear mechanical defects and functional abnormalities associated with progeria.

### Progerin density is spatially more heterogeneous than the WT lamin A/C distribution along the nuclear envelope

**Fig. 4.**
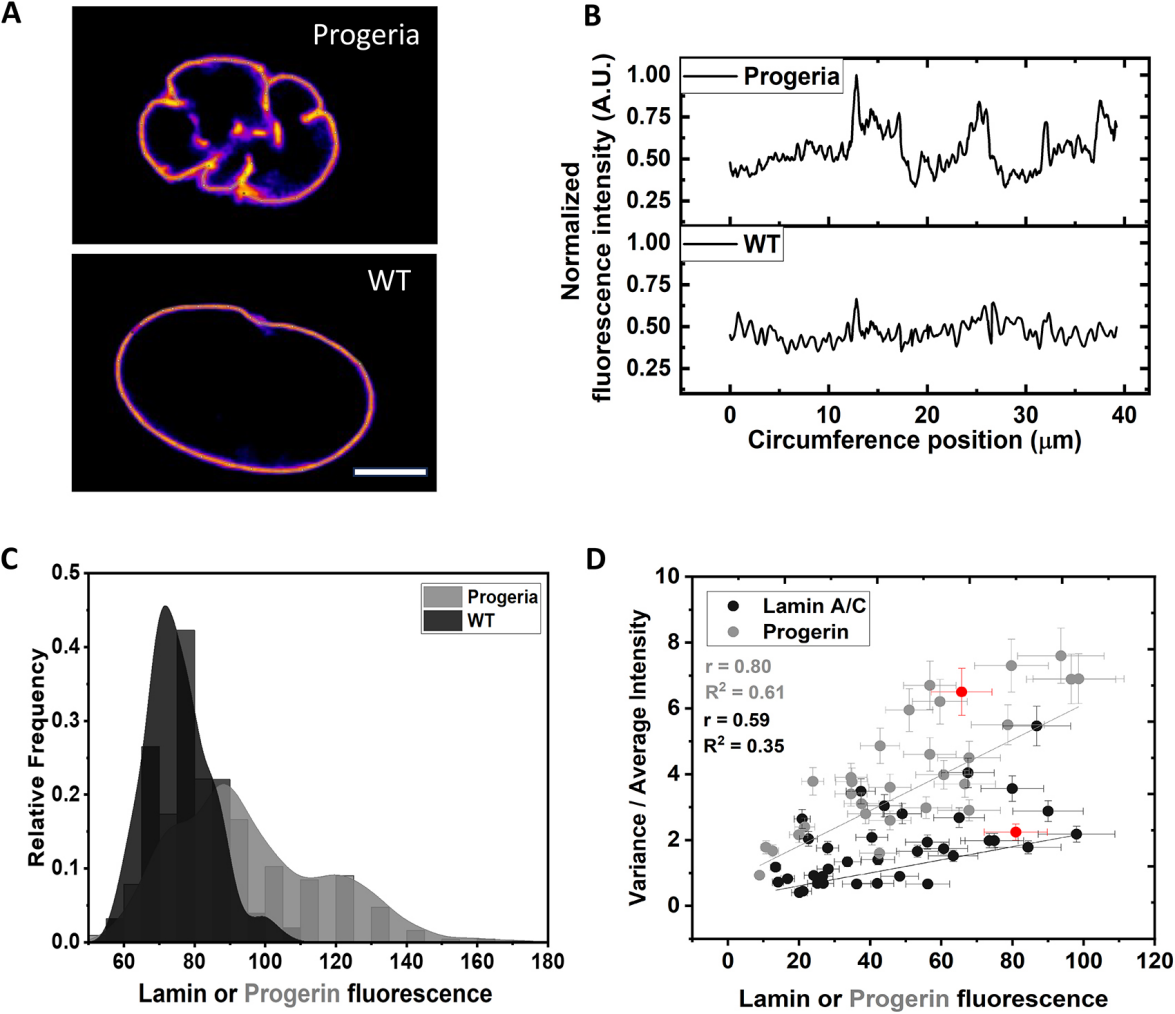
**Increased heterogeneity in progeria cells.** (A) Representative images showing progerin fluorescence in progeria cells (top) and lamin A/C fluorescence in WT cells with a yellow line marking the 3-pixel-wide ROI used for the intensity profile in B. Scale bar: 5 μm. (B) Quantification of image in A shows a higher degree of heterogeneity in the progeria nucleus compared to the WT nucleus. (C) Histogram showing narrow and high peaks for local lamin A/C fluorescence compared to short and broad peaks for progerin fluorescence in A. (D) Both progeria and WT cells show increased heterogeneity of progerin or lamin A/C distribution with an increase in protein concentration, but heterogeneity in progeria was found to increase more with overall expression (*n*≥32 for each cell type from three independent experiments; error bars represent measurement error; red datapoints indicate respective WT and progeria cell in A). ANCOVA revealed a significantly steeper slope of spatial heterogeneity as a function of protein expression in progeria compared to WT (t=−3.282, d.f.=63, p=0.002).

The spatial distributions of progerin and lamin A/C along the nuclear envelope are not uniform (Fig. 4A). This heterogeneity is significantly pronounced in progeria cells, where the persistent farnesylation of progerin leads to abnormal accumulation and clustering at discrete regions of the nuclear membrane, as revealed by fluorescence intensity profiles along the 3-pixel line at the nuclear membrane normalised to their respective maximum intensity values (Fig. 4B). The histogram of lamin A/C fluorescence intensities along the nuclear circumference in WT cells reveals that it had a relatively narrow distribution, suggesting a uniform density of lamin A/C throughout the nuclear envelope. In contrast, progeria cells exhibited a broadened and flattened fluorescence intensity distribution, indicating that progerin density is distributed more heterogeneously along the nuclear lamina (Fig. 4C). Quantitative analysis showed that this spatial inhomogeneity increased with overall lamin expression in both WT and progeria cells. However, progeria cells displayed a markedly higher sensitivity to expression levels, as evidenced by a steeper rise in the variance of local progerin fluorescence intensity relative to total expression (Fig. 4D). This suggests that the integration of progerin into the lamina is inherently more disordered compared to lamin A/C, likely due to its abnormal post-translational modification and aggregation propensity. The rate of increase in spatial heterogeneity with expression was significantly greater in progeria than in WT cells (ANCOVA, t=−3.282, d.f.=63, p=0.002), consistent with the inherently more disordered integration of progerin into the nuclear lamina.

Spatial heterogeneity in lamina protein distribution directly translates into local variations in nuclear membrane stiffness, influencing the mechanical response and functional properties of the nucleus (Srivastava et al., 2021). Under mechanical stress, these heterogeneous regions might govern localised deformation and strain distribution across the nuclear envelope. Previous studies have linked nuclear membrane strain to nuclear pore opening dynamics (Zimmerli et al., 2021), which modulate nucleocytoplasmic transport of transcriptional regulators and other mechanosensitive factors. Therefore, the enhanced spatial heterogeneity of progerin in progeria could disrupt nuclear shuttling processes, potentially impacting gene expression and cellular function.

### Local lamin density regulates local nuclear deformation along the nuclear membrane by modulation of the chromatin state of the nucleus

**Fig. 5.**
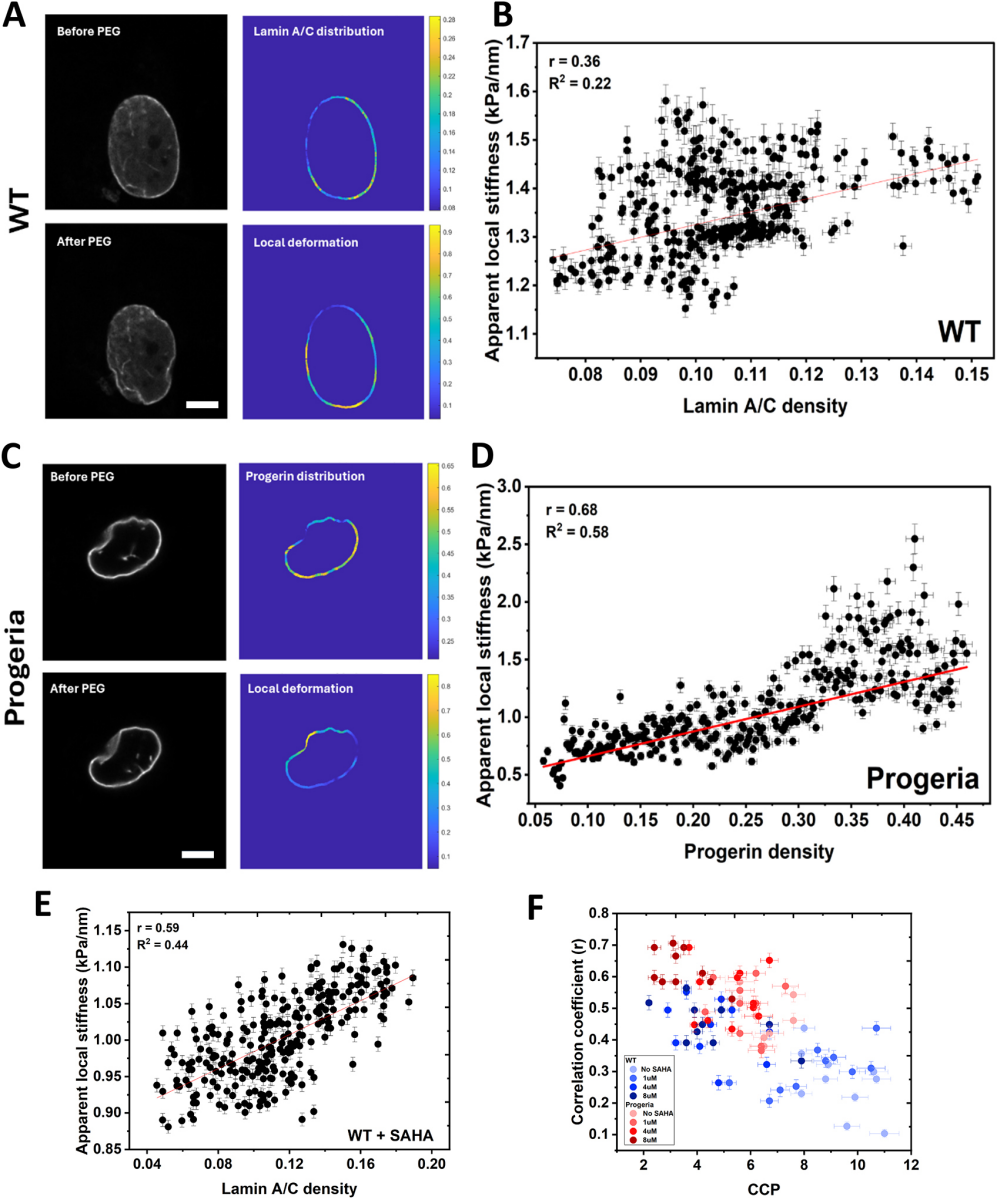
**Strain mapping in progeria and WT cells.** (A) Representative WT nucleus before compression (top left) and after osmotic compression (bottom left; 2.5% PEG, ΔP=193.6 kPa), with corresponding lamin A/C distribution map (top right) and local deformation map (bottom right). Scale bar: 5 μm. (B) Correlation between lamin A/C density and apparent local stiffness in WT cells (Pearson r=0.36; error bars represent measurement error). (C) Representative progeria nucleus before compression (top left) and after osmotic compression (bottom left), with corresponding progerin distribution map (top right) and local deformation map (bottom right). Scale bar: 5 μm. (D) Stronger correlation between progerin density and apparent local stiffness in progeria cells compared to WT (Pearson r=0.68; error bars represent measurement error). (E) The correlation between lamin A/C density and local nuclear stiffness increases after chromatin decondensation using SAHA in WT cells (Pearson r=0.59). Plots in B, D and E are representative plots of a single cell showing local stiffness and protein density along the nuclear membrane. (F) The correlation coefficient between local nuclear stiffness and corresponding local lamin A/C or progerin density increases with increasing SAHA concentration in both WT (blue) and progeria (red) cells, but untreated progeria cells show a consistently higher correlation at each concentration due to reduced baseline heterochromatin (*n*=40 nuclei for each cell type from two independent experiments; error bars represent measurement error).

An inverse correlation between local lamin A/C density and local deformation under osmotic stress, suggesting that lamin A/C density determines local nuclear stiffness, has been previously reported (Srivastava et al., 2021). This is exemplified in Fig. 5, where panel A shows a representative WT nucleus before compression (top left) and after osmotic compression (bottom left; 2.5% PEG; net pressure=193.6 kPa), alongside the corresponding lamin A/C distribution map (top right) and local deformation map (bottom right), indicating more significant nuclear deformation in regions with low lamin A/C density and vice-versa. The equivalent analysis for progeria cells is shown in Fig. 5C, with the nucleus before compression (top left) and after compression (bottom left), alongside the progerin distribution map (top right) and local deformation map (bottom right), confirming that regions with low progerin density display greater local deformation. Subsequently, we noted a positive association between the local nuclear stiffness and the lamin A/C density in WT cells, as shown in Fig. 5B with a Pearson's correlation coefficient of 0.36. A stronger positive association was observed between local nuclear stiffness and progerin density in progeria cells, as shown in Fig. 5D with a Pearson's correlation coefficient of 0.68, which was higher than that for WT nuclei. If progerin or lamin A/C were solely responsible for nuclear stiffness, we would anticipate a correlation coefficient of 1, and if it played little role in nuclear stiffness, we would expect a coefficient value close to 0. This suggests that progerin density plays a larger role in progeria local nuclear mechanics than lamin A/C does in WT nuclei.

Another key factor which plays a crucial role in nuclear stiffness is the chromatin within the nucleus (Stephens et al., 2017). Thus, the correlation coefficient in this context acts as a metric that reflects both the nuclear mechanics and the chromatin state of the cell. The rise in correlation coefficient in the context of progeria is possibly due to the inherent depletion of peripheral heterochromatin markers (Shumaker et al., 2006; Chojnowski et al., 2020) thus making local deformation more correlated with the progerin density at the nuclear membrane. Previous studies have shown that nuclear deformation triggers the translocation of enzymes, such as histone deacetylase 3 (HDAC3), which increase heterochromatin markers (Damodaran et al., 2018). This would imply that stiffer progeria nuclei inherently have reduced activity of HDAC3, which is a possible explanation for reduced heterochromatin in progeria cells. Next, to examine a more direct role of chromatin condensation on the correlation between lamin A/C density and local nuclear deformation, we first treated the WT cells with suberoylanilide hydroxamic acid (SAHA; a histone deacetylase inhibitor that promotes chromatin decondensation by preventing removal of acetyl groups from histone tails) (Marks et al., 2001) (see Fig. S4) and this resulted in an increase in Pearson's correlation coefficient as shown in Fig. 5E (r=0.59). We also treated the cells with different concentrations of SAHA, which decondenses chromatin, and quantified the degree of chromatin condensation in terms of the chromatin condensation parameter (CCP) (Irianto et al., 2014). This was followed by strain mapping using fast iterative digital volume correlation (FIDVC) (Bar-Kochba et al., 2015) in WT and progeria cells, as described in detail in the methods section. The correlation coefficient between lamin A/C or progerin density and local nuclear stiffness increased with greater concentrations of SAHA in both progeria and WT cells, indicating that the chromatin condensation state influences nuclear mechanics. However, untreated progeria cells showed lower CCP values due to a lower proportion of heterochromatin markers inherently present (Fig. 5F). This suggests a stronger dominance of local nuclear deformation on progerin density as compared with the WT lamin A/C.

### Progerin-induced nuclear stiffening impairs mechanosensation

**Fig. 6.**
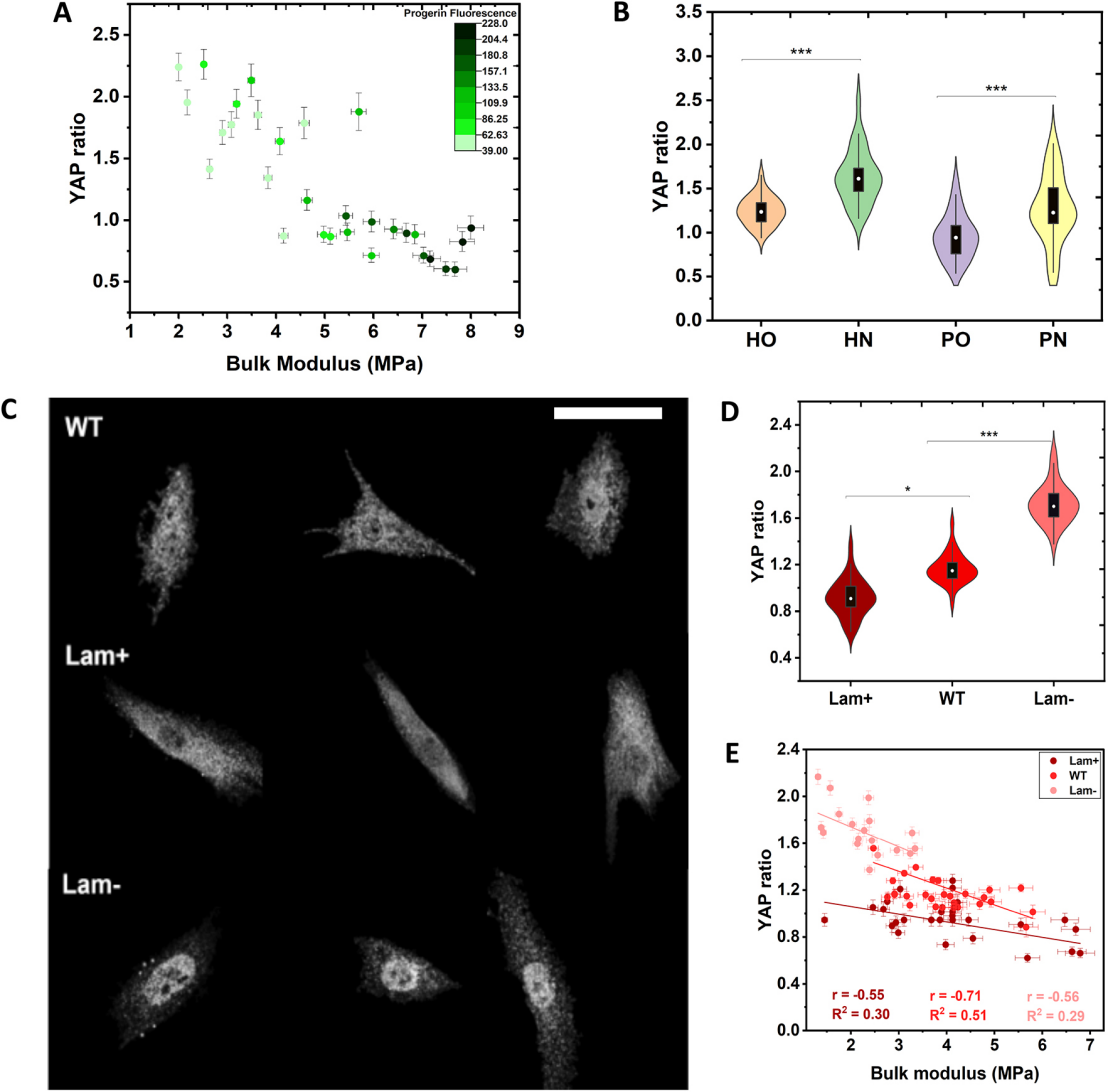
**Reduced nuclear YAP translocation in stiffer nuclei.** (A) The YAP ratio was found to reduce with increase in nuclear stiffness quantified as bulk modulus (*n*=30 nuclei from 2 independent experiments; error bars represent measurement error). (B) The nucleo-cytoplasmic YAP ratio was found to be highest in the youngest HN cells (passage 7), followed by very similar PN (passage 7) and HO (passage 22) and finally, the oldest PO cells (passage 22) (*n*≥28 for each cell type from three independent experiments). HN, healthy new WT; HO, healthy old WT; PN, progeria new; PO, progeria old. ***P<0.001 (one-way ANOVA with Tukey's post hoc test). (C) Example nuclei of WT cells, lamin A/C overexpressing WT cells (Lam+) and lamin A/C, silenced WT cells (Lam−) stained with YAP antibody. Scale bar: 50 μm. (D) The YAP nucleo-cytoplasmic ratio was found to be maximum for the more compressible Lam− cells, followed by WT and the lowest in the least compressible Lam+ cells nuclei (*n*≥30 for each cell type from three independent experiments). ***P<0.05 (one-way ANOVA with Tukey's post hoc test). (E) YAP ratio decreases in all cells with increasing bulk moduli (*n*≥30 for each condition from three independent experiments; error bars represent measurement error). Data in B and E shown as violin plots with a box plot in the middle highlighting median (centre dot), interquartile range (box), and 1.5× IQR from the Q1 and Q3 boundaries (whiskers).

Having established that progerin cross-linking leads to nuclear stiffening in progeria cells, we next investigated whether these mechanical alterations impair the ability of the nucleus to sense and respond to mechanical cues. To this end, we utilised YAP, a downstream effector of the Hippo signalling pathway and a well-characterised mechanosensitive transcriptional regulator, as a functional readout of nuclear mechanosensitivity. We quantified the nuclear-to-cytoplasmic ratio of YAP (YAP ratio) in fibroblasts across a range of progerin expression levels and corresponding nuclear stiffness. Progeria nuclei, characterised by elevated bulk modulus, consistently exhibited reduced YAP nuclear localisation (Pearson r=−0.670, p<0.001; Fig. 6A).

Population-level analysis further demonstrated that nuclear YAP localisation declined with both cellular ageing and nuclear stiffness. The YAP ratio was highest in the youngest (or newest) wild-type fibroblasts (HN, passage 7; 1.63±0.31), decreased in normally ageing wild-type cells (HO, passage 22; 1.25±0.16). The progeria cells showed a similar trend with higher YAP nuclear/cytoplasmic ratio in early-passage progeria fibroblasts (PN, passage 7; 1.27±0.39) and were found to be the lowest in late-passage progeria cells (PO, passage 22; 0.95±0.26) (mean±s.d.; Fig. 6B). Thus, both normal cellular ageing and progerin-induced premature ageing suppress YAP nuclear localisation in a stiffness-dependent manner. To test whether this defect reflects a more general mechanical principle beyond progerin pathology, we experimentally modulated lamin A/C levels in WT cells. Overexpression of lamin A/C (Lam+) reduced YAP nuclear translocation, whereas lamin-depleted cells (Lam−) displayed enhanced YAP localisation (Fig. 6C,D). Importantly, across these experimentally manipulated conditions, nuclear stiffness exhibited a robust inverse correlation with the YAP nuclear-to-cytoplasmic ratio (Fig. 6E), indicating that nuclear stiffening directly impairs mechanosensitive transcription factor import.

This relationship establishes nuclear stiffness as a key determinant of mechanosensitive transcription factor localisation, independent of the specific molecular mechanism driving lamina stiffening. Collectively, these findings demonstrate that pathological stiffening of the nuclear lamina, as observed in progeria, blunts the ability of the nucleus to sense and transmit mechanical signals. By impeding YAP nuclear translocation – a critical regulatory protein – these mechanical alterations disrupt mechanotransduction-dependent transcriptional responses, contributing to gene expression dysregulation and cellular dysfunction in laminopathies. The functional readouts highlighted in Fig. 6 thus establish a direct mechanistic link between nuclear architectural defects and impaired mechanical signal interpretation at the transcriptional level. The observed chromatin decompaction and persistent lamina wrinkling in progeria cells further support the hypothesis that multiple pathways sensitive to nuclear mechanics might be disrupted, warranting future investigations into broader transcriptional dysregulation arising from nuclear stiffening.

## DISCUSSION

Recent advances have highlighted the central role of nuclear mechanics in regulating cellular function and its implications in various pathologies, including malignancies and laminopathies. Our study demonstrates that nuclear stiffening in HGPS arises not only from elevated progerin expression but crucially from enhanced cross-linking of progerin within the nuclear lamina. This stiffening is further exacerbated by the formation of intranuclear progerin aggregates, which mechanically tether to the nuclear envelope, contributing to persistent wrinkling and lamina distortion.

To quantitatively capture how lamin cross-linking impacts nuclear stiffness, we developed the CLEF. Although the CLEF was derived and validated in progeria nuclei, where persistent progerin farnesylation exaggerates cross-linking, its conceptual framework is broadly applicable to other contexts where lamina dynamics are altered, such as in other laminopathies, and upon stress-induced lamina remodelling or oncogenic transformations. As a generalisable predictive parameter, the CLEF could help assess nuclear mechanics and how altered lamin cross-linking modulates mechanotransduction and cellular function. Although our study was performed in fibroblast cultures to dissect the biophysical mechanisms of progerin-induced nuclear stiffening, the implications are likely relevant *in vivo*, including vascular tissues or stem cell niches. Future work employing organotypic cultures or animal models could validate how lamina cross-linking-driven nuclear rigidity impacts tissue architecture and systemic mechanotransduction. Further analysis reveals a more heterogeneous distribution of progerin in progeria nuclei compared to that for the WT lamin A/C. This heterogeneity correlates more strongly with local nuclear deformation in progeria, possibly due to reduced peripheral heterochromatin (Shumaker et al., 2006; Chojnowski et al., 2020). Chromatin decondensation in WT cells led to a stronger correlation between lamin A/C density and local stiffness, supporting the idea that chromatin compaction contributes to local nuclear mechanics.

These findings offer insight into mechanosensory dysfunction in progeria, where abnormal nuclear architecture impairs mechanical signal processing and alters gene expression (Najdi et al., 2021; Martin et al., 2022). Increased nuclear stiffness and heterogeneous protein distribution likely contribute to disrupted responses in pathways regulating cell differentiation, migration and survival. The link between nuclear stiffness and chromatin reorganisation suggests that mechanical changes might modulate gene expression in disease contexts.

Lamin A regulates both local and overall nuclear stiffness, which can influence nuclear pore function. Previous studies have shown that more deformable nuclei tend to have more open nuclear pores (Kalukula et al., 2022; Elosegui-Artola et al., 2017). In progeria, increased stiffness might impair pore mechanics, potentially altering molecular trafficking and transportation of transcription factors like YAP. Understanding this relationship might clarify how nuclear structural changes impact downstream gene regulation and related physiological consequences.

Functionally, these mechanical alterations significantly impair nuclear mechanosensation. We demonstrate that increased nuclear stiffness correlates with diminished nuclear translocation of YAP, a key mechanosensitive transcription factor. This inverse relationship persists across various cellular manipulations affecting lamina cross-linking and stiffness, establishing nuclear rigidity as a direct modulator of mechanotransduction. These results provide a mechanistic explanation for the transcriptional dysregulation observed in laminopathies, where structural defects at the nuclear envelope propagate into impaired cellular responses to mechanical stimuli. Although YAP was employed here as a representative mechanosensitive transcription factor, similar stiffening-induced impairments could extend to other mechanotransducers such as MRTF-A (Gupta et al., 2013) or HDAC3 (Damodaran et al., 2018). It is important to note that the present study reports YAP nuclear localisation as a readout of mechanosensory activity, consistent with established practice in the field. Direct quantification of YAP target gene expression (e.g. that of CTGF or CYR61) alongside investigation of MRTF-A and HDAC3 would be required to confirm downstream transcriptional consequences, and these are priorities for future investigation.

In summary, our work elucidates how abnormal lamina cross-linking and intranuclear progerin tethering stiffen the nucleus and disrupt mechanosensitive signalling. By establishing a quantitative framework linking nuclear architecture to mechanical resilience and signal transduction, these findings advance our understanding of how structural defects at the nuclear envelope contribute to cellular dysfunction in laminopathies. Targeting lamina cross-linking dynamics and restoring nuclear adaptability might represent viable therapeutic strategies to mitigate mechanosensory dysfunction in progeria and related disorders.

## MATERIALS AND METHODS

### Cell culture and modulating chromatin condensation

Wild-type human dermal fibroblasts (Coriell Institute, cat. #GM03234; male donor, age 23 years) and HGPS dermal fibroblasts (Coriell Institute, cat. #AG11498; male donor, age 14 years) were cultured following standard ATCC protocols in Eagle's minimum essential medium (EMEM) supplemented with 15% FBS and 1% penicillin-streptomycin antibiotic (all Wisent) (Mao et al., 2020). Cell lines were authenticated by the supplier and routinely tested for mycoplasma contamination; all cultures were confirmed to be contamination-free. For comparative analyses, cells were subdivided into early-passage (‘new’, passage 7) and late-passage (‘old’, passage 22) groups. These are referred to in the Results and Fig. 6 as HN (healthy new WT), HO (healthy old WT), PN (progeria new) and PO (progeria old). The degree of chromatin condensation was modified by altering the charge on the histone complex by inhibiting the enzyme HDAC using different dosages (1 μM, 2 μM and 8 μM) of SAHA (Cayman Chemical) (Marks et al., 2001; Hockly et al., 2003) for 20 h. The chromatin state was quantified using the commonly used metric CCP (Irianto et al., 2014), which characterises chromatin condensation based on the fluorescence intensity (Hoechst 33342 stained; Thermo Fisher Scientific) and the total area covered by heterochromatin foci as a fraction of the total nuclear area. For silencing experiments, endogenous lamin A/C was knocked down using an RFP-tagged inducible shRNA construct targeting lamin A/C (Dharmacon, cat. #V3THS_352819). Silencing was induced 24 h prior to transfection with either mCherry-tagged wild-type lamin A/C (Addgene plasmid #55068, deposited by Michael Davidson) or EGFP-tagged lamin A D50 mutant (Addgene plasmid #17653, deposited by Tom Misteli), and cells were imaged 24 h post-transfection. For lamin A/C overexpression experiments (Fig. 6), WT cells were transfected with mCherry-lamin A/C (plasmid as above) without prior silencing and imaged 24 h post-transfection.

### Quantification of nuclear bulk moduli

The cells were attached to fibronectin-coated coverslips. Then the cells were exposed to medium supplemented with 10% (w/w) 400 Da polyethylene glycol (PEG 400, Sigma), thus, exerting 1514 kPa osmotic stress for 20 min to reach an equilibrium of compression (Zhou et al., 2009). The osmotic pressure values were adopted from Khavari and Ehrlicher (2019), in which osmolality of PEG 400 solutions was measured by freezing-point depression osmometry and converted to pressure via Π=osmolality×R×T (R=8.314 J mol⁻¹ K⁻¹, T=310 K). The isotonic medium baseline is P₀=743 kPa (∼288 mOsm). For PEG-supplemented medium: P₁=936.6 kPa (2.5% w/w PEG 400) and P₁=1514 kPa (10% w/w PEG 400), giving ΔP=193.6 kPa and 771 kPa respectively.

Cell nuclei were labelled with Hoechst 33342. Confocal microscopy *xyz*-stacks (63× objective/1.4 NA, Leica SP8) were acquired before and after compression. Nuclear volumes were measured as described below. The cells compress due to the hypertonic osmotic pressure, causing an efflux of water (Guo et al., 2017). This volume change is measured and used to determine the bulk modulus the relationship B=−ΔP/(ΔV/V) where B is bulk modulus, ΔP is osmotic pressure, ΔV is change in volume, and V is original volume (Khavari and Ehrlicher, 2019). In general, for all our image processing, data integrity was maintained by excluding cells that exhibited significant translational drift or moved out of the imaging frame upon the addition of PEG.

### Calculation of intranuclear lamin irregularity

To measure lamin heterogeneity, we developed a MATLAB-based analysis. For each nucleus, *z*-stacks were converted into a sum-of-slices projection and segmented by adaptive thresholding with basic morphological cleanup. We then generated a radial profile by averaging fluorescence in concentric 1-pixel rings moving inward from the nuclear rim (0.10 μm per step) and normalised each profile to its maximum. To correct for the natural decrease in intensity toward the centre, we subtracted a smooth baseline obtained with moving filters. The ‘interior’ region for analysis began at the first local minimum ≥0.4 μm inside the rim and extended to 95% of the nuclear radius (expanded if too narrow). Finally, we defined interior variance (IV) as the variance of the baseline-subtracted signal within this interior region, providing one value per nucleus for group comparisons.

### Quantification of nuclear WI

Mauck's laboratory developed a macro for ImageJ that was used here to quantify the wrinkling index (WI; Cosgrove et al., 2021). The macro employs *z*-stacks of lamin-A/C (or progerin) immunostained nuclei with a pixel size of 100 nm/pixel and a *z*-stack height of 300 nm/*z*-slice. It is essential to have this high image resolution to offer sufficient contour boundaries for the WI computation. In brief, the macro generates a maximum projection picture of the z-stack, auto-contrasts it, and converts it to a mask. Next, the maximum projection image was binarised using the FeatureJ edge tracking algorithm in ImageJ, and the information for minimum/maximum/mean/median intensity, standard deviation, and nuclear aspect ratio was recorded. The binarised output of the FeatureJ algorithm was converted into a ‘wrinkle index’ by computing the mean bright pixel intensity, dividing by 255 (the highest 8-bit white pixel intensity), and multiplying by 100 to get a percentage of nuclear spread area covered by wrinkled outlines.

### 3D volume measurement of nuclei

The nuclear volume was calculated as described previously (Srivastava et al., 2021). Briefly, *xyz* stacks of nuclei stained with Hoechst 33342 were imaged using a 63×/1.4 NA oil immersion objective on a Leica SP8 confocal microscope with a *z*-step size of 0.3 μm, and an *xy* resolution of 0.17 μm/pixel. 3D visualisation and measurement were performed using ImageJ software.

### Nuclear strain mapping

To measure local strain on the nuclear membrane, *z*-stack images of nuclei labelled with GFP Chromobody (Chromtek, cat. no. lcg) for lamin A/C or GFP–progerin (pEGFP-Δ50 lamin A) were acquired immediately before and after exposure to 2.5% PEG 400 (ΔP=193.6 kPa) with a *z*-step size of 300 nm. Images were passed to a customised MATLAB implementation of the FIDVC algorithm (Bar-Kochba et al., 2015), which tracks sub-pixel displacements by FFT-based cross-correlation within overlapping 32-pixel (1.86 μm) subset windows, iteratively refined to sub-pixel accuracy. The output is a full 3D displacement vector field from which the complete strain tensor including normal strains E_xx_, E_γγ_, E_rr_, shear strains, hydrostatic and von Mises strain is computed from spatial gradients across the entire nuclear volume. For the lamin or progerin density versus apparent stiffness analysis, displacement magnitude is extracted at the nuclear membrane ring at the mid-nuclear *z*-slice. To reduce sub-pixel tracking noise while retaining biological resolution, ring pixels are grouped into non-overlapping 16-pixel bins (0.93 μm), satisfying the Nyquist criterion for statistically independent estimates between adjacent bins. Local apparent stiffness is computed as K=ΔP/u (kPa/nm) per bin, where ΔP=193.6 kPa is the net applied osmotic pressure and u is the mean displacement magnitude within that bin. K represents a normalised mechanical resistance rather than an intrinsic elastic modulus, analogous to a spring constant defined as force per displacement. Spatial variation in K therefore directly encodes spatial variation in local mechanical resistance under a controlled, experimentally invariant loading condition. Representative images before and after compression and strain maps are shown for both progeria and WT nuclei in Fig. 5.

### Western blotting

To perform western blot, first, the proteins were extracted using RIPA buffer (1% Triton X-100, 0.5% sodium deoxycholate, 0.1% SDS and 1 mM EDTA). The concentration of isolated proteins was measured (Nanodrop). For western blot analysis, the proteins were separated based on size via SDS-PAGE using a pre-cast gradient gel (Bio-Rad). The proteins were transferred to nitrocellulose membranes, followed by blocking the membrane with 5% BSA overnight. The membranes were then incubated with primary antibodies specific to lamin A/C protein (1:500; NEB catalog # 2032S). After washing the membrane with the running TBST (10 mM Tris-HCl, pH 8.0, 150 mM NaCl, 0.05% Tween 20) buffer, the membranes were incubated with secondary antibodies conjugated with horseradish peroxidase (Bio-Rad). The protein bands were visualised using ChemiDoc (Bio-Rad).

### Fluorescence recovery after photobleaching

FRAP experiments were performed to assess the mobility of lamin A or progerin in live WT and progeria cells. Cells transfected with mCherry-tagged wild-type lamin A/C or EGFP-tagged lamin A D50 mutant plasmid were plated on glass-bottom dishes and maintained in Phenol Red-free imaging medium during imaging. All experiments were conducted at 37°C.

A region of interest (ROI) at the nuclear membrane was selected, and 100 pre-bleach frames were acquired at an interval of 0.26 s to establish baseline fluorescence. Photobleaching was performed using a high-intensity laser pulse (100%) targeted at the ROI. Following bleaching, fluorescence recovery was monitored by acquiring time-lapse images at low laser intensity at 0.26 s interval until a recovery plateau was reached after 200 s.

Fluorescence intensities were quantified using ImageJ. Data were normalised to prebleach values and corrected for overall photobleaching and background fluorescence. Recovery curves were fitted using a single exponential model to extract mobile fractions using the equation *F*(*t*)=*F*_∞_​−(*F*_∞_​−*F*_0_​)⋅e^−kt^, where *F*(*t*) is fluorescence at time *t*, *F*_0_​ is fluorescence immediately after bleaching, *F*_∞_​ is the plateau fluorescence, and *k* is the recovery rate constant (Axelrod et al., 1976).

### Cross-linked lamin expression factor

The CLEF was defined as the product of mean lamin or progerin fluorescence intensity per nucleus (measured as mean fluorescence along the nuclear rim ring) and the immobile fraction derived from FRAP. CLEF=fluorescence intensity×immobile fraction. This composite metric captures both protein abundance and the extent of lamina cross-linking.

### Nucleo-cytoplasmic YAP and immunofluorescence calculation

A custom-written MATLAB code was used to calculate the YAP nucleo-cytoplasmic ratio (YR) as employed in diverse studies (Koushki et al., 2023). Briefly, the code creates a mask for the nuclei based on Hoechst 33342 staining and a mask for the entire cell based on YAP immunofluorescence. The mean fluorescence is calculated for the nuclear region and the cytoplasmic (excluding the nucleus) region of cells stained with anti-YAP fluorescence antibodies (see below), followed by dividing the nuclear fluorescence with the cellular fluorescence to obtain the nucleo-cytoplasmic ratio.

### Quantification of chromatin condensation parameter

To quantify chromatin condensation state, nuclei were stained with Hoechst 33342 (Thermo Fisher Scientific) for 10 min at room temperature. *Z*-stack images were acquired using a 63×/1.4 NA oil immersion objective on a Leica SP8 confocal microscope and maximum intensity projections were generated. A MATLAB-based pipeline adapted from Irianto et al. was used for quantification (Irianto et al., 2014). Briefly, the nuclear boundary was segmented by thresholding of the Hoechst channel. The Sobel edge detection algorithm was applied within the nuclear mask to detect chromatin domain boundaries, and the CCP was computed as the total edge density within the nucleus. Higher CCP values reflect a greater degree of chromatin condensation with more distinct domain boundaries; lower values reflect decondensed, more homogeneous chromatin.

### Immunostaining

Cells were fixed in 4% paraformaldehyde (PFA) in PBS for 10 min at room temperature, followed by three washes with PBS. Samples were permeabilised with 0.2% Triton X-100 in PBS for 10 min and then blocked in 1% bovine serum albumin (BSA) in PBS supplemented with 0.2% Triton X-100 (PBST) for 45 min.

Samples were subsequently incubated overnight at 4°C with primary antibodies against lamin A/C (1:500; Thermo Fisher Scientific, MA5-35284) (Neely et al., 2023) or progerin (1:500; Santa Cruz Biotechnology, sc-81611) (Glynn and Glover, 2005) or YAP (1:1000; Thermo Fisher Scientific, #66900-1-Ig) (Guo et al., 2023). Antibody specificity was based on manufacturer-provided validation data.

After three washes in PBST, samples were incubated with species-specific secondary antibodies (1:500 in blocking buffer; Thermo Fisher Scientific) for 1 h at 37°C in the dark. Secondary antibodies included anti-rabbit IgG or anti-mouse IgG conjugated to appropriate fluorophores.

### Statistical analysis

The sample size in our study was determined based on the technical and temporal requirements of maintaining long-term cell cultures for over 6 weeks at times. Given the focus on cellular ageing, a random sampling approach was used to select representative replicates from independent culture batches. This ensured that the data captured the biological variability inherent in the ageing process while maintaining the feasibility of the longitudinal experimental design. Data are presented as mean±s.d. unless otherwise indicated. For comparisons involving more than two groups, one-way ANOVA followed by Tukey's post hoc test was applied to evaluate pairwise differences while correcting for multiple comparisons. Pairwise group comparisons are indicated in the text and figure legends. Statistical significance is reported as *P<0.05; **P<0.01; ***P<0.001. Scatter plots include Pearson correlation coefficients (r) and coefficients of determination (R²) derived from linear regression, reported per group where applicable. Also, where applicable, comparison of regression slopes between groups was performed by ANCOVA to test for differences in the rate of association between variables across conditions, with significance assessed at *P*<0.05. Comparison of Pearson correlation coefficients between independent groups was performed using Fisher's z-transformation, with |z|>1.96 taken as the threshold for statistical significance at *P*<0.05 (two-tailed). Population-level data (multiple independent measurements per condition) are reported as mean±s.d. For single-cell-based readouts where only one value is obtained per cell, error bars denote propagated measurement error from image analysis rather than variability across replicates.

### Use of AI tools

ChatGPT (OpenAI) and Claude (Anthropic) were employed to rephrase and polish sections of the text for clarity. All outputs were critically reviewed, edited and verified by the authors to ensure accuracy and integrity of the scientific content.

## Supplementary Material

10.1242/joces.264519_sup1 Supplementary information
