# Supplementary material for: Progerin cross-linking stiffens the nucleus and impairs mechanosensation in Hutchinson–Gilford progeria syndrome
Source: J Cell Sci. 2026 Jun 19;139(12):jcs264519. doi: 10.1242/jcs.264519 (PMC13327536; doi:10.1242/jcs.264519)
Supplement: Supplementary information [file joces-139-264519-s1.pdf]

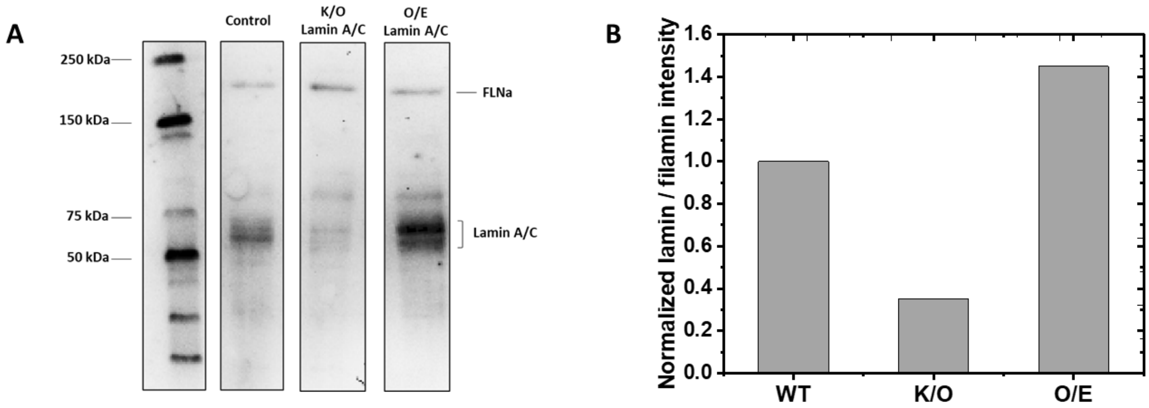

Fig. S1. Western blot analysis: A, Western blot showing faint lamin A/C bands in lamin A/C silenced cells and dense bands in lamin A/C overexpressed cells. **B,** Quantitative analysis of western blot showing maximum expression in lamin A/C overexpressed cells and least in lamin A/C silenced cells.

Journal of Cell Science • Supplementary information

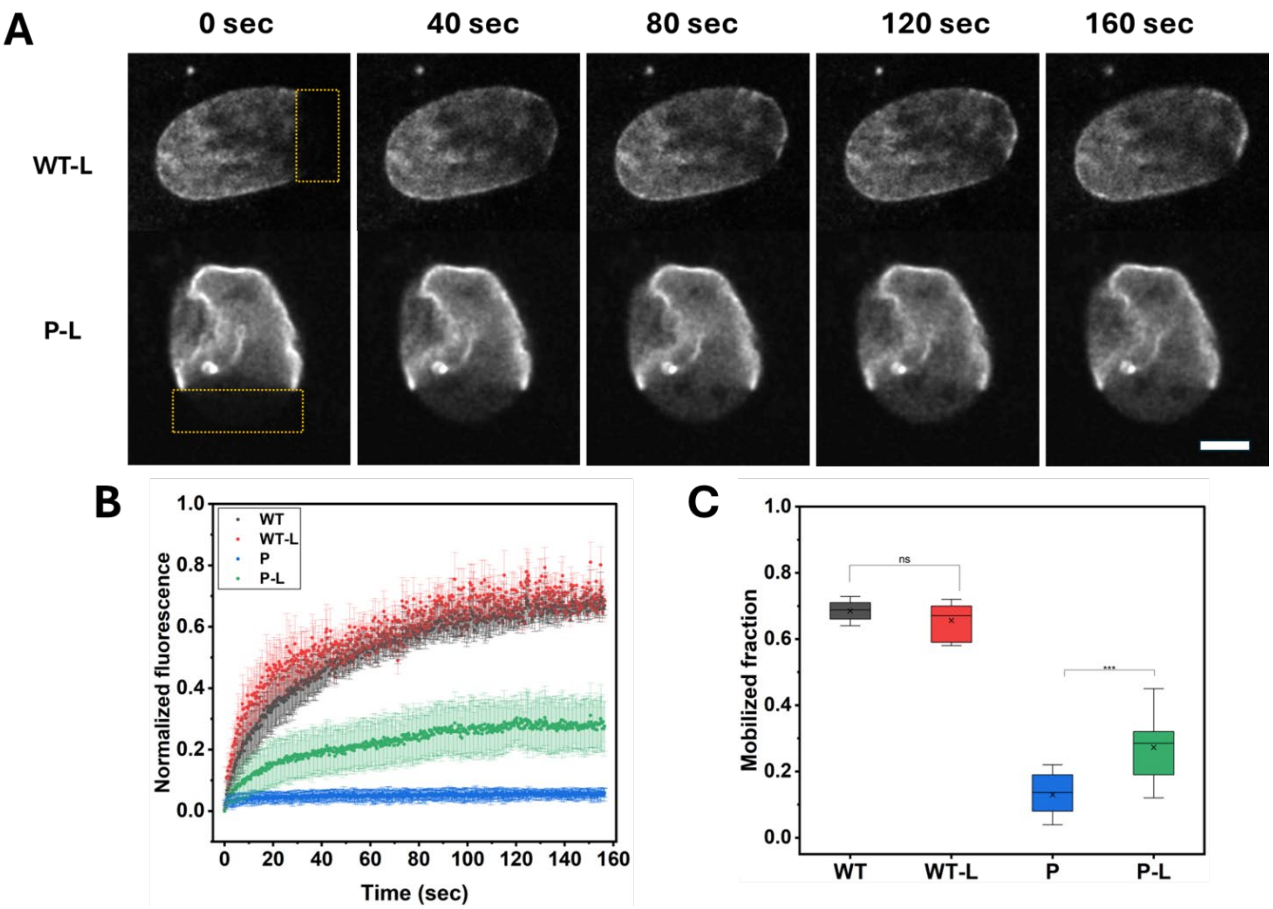

Fig. S2. Effect of lonafarnib in progerin cross-linking: A, Representative FRAP time-lapse images of WT cells treated with lonafarnib (WT-L, top) and progeria cells treated with lonafarnib (P-L, bottom) (scalebar = 6 µm). **B**, FRAP recovery curves for all four conditions: WT (grey), WT-L (red), P (blue), and P-L (green), showing that lonafarnib increases progerin recovery in progeria cells but does not significantly alter WT lamin A/C dynamics. **C**, Mobilized fraction across all four conditions; lonafarnib does not significantly change WT mobility (ns) but significantly increases progerin mobility (***) (n = 30 nuclei per condition from 3 independent experiments; mean ± SD)

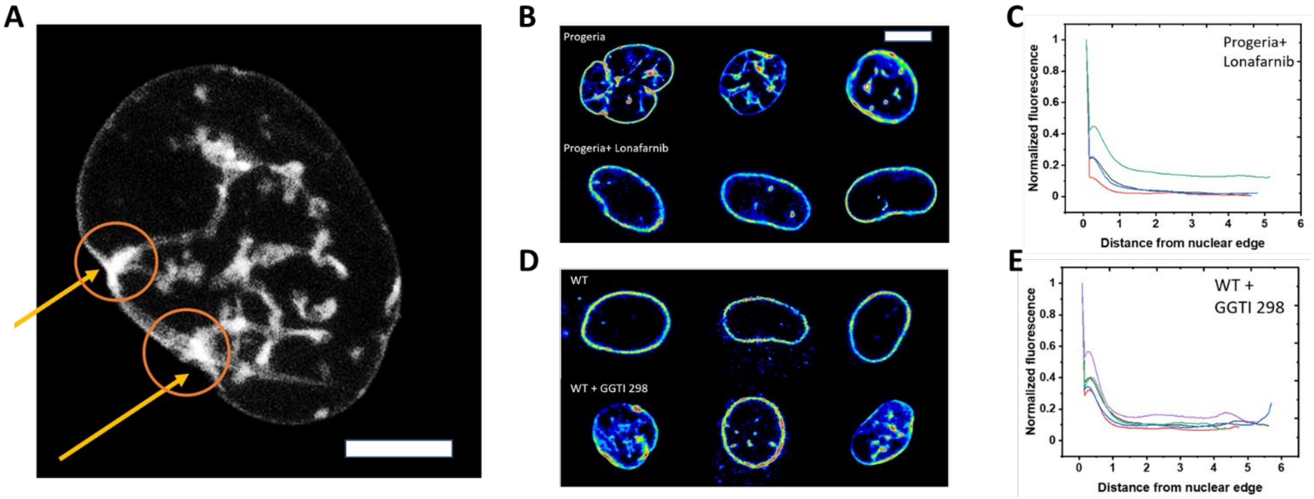

Fig. S3. Nuclear wrinkling in Progeria cells A, Progerin structures pull the nuclear membrane inwards (scalebar = 6µm). **B,** Representative Progeria nuclei showing reduced internal structures after lonafarnib treatment (scalebar = 5µm). **C,** Quantification of normalized mean fluorescence from edge to inside showing homogenous fluorescence inside the membrane indicating lack of internal structures after lonafarnib treatment. **D,** Representative WT fibroblasts nuclei showing an increase in internal structures after GGTI treatment. **E,** Quantification of normalized mean fluorescence from edge to inside showing an increase in heterogeneity in mean fluorescence inside the membrane indicating development of internal structures after GGTI treatment.

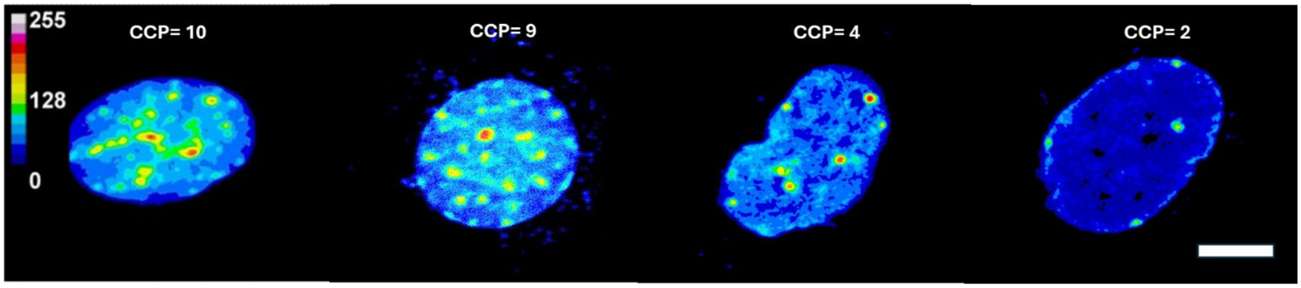

Fig. S4. Chromatin condensation parameter A, Hoechst 33342 stained nuclei showing varied degree of chromatin condensation along with their CCP

Journal of Cell Science • Supplementary information
